# A Qualitative Description of Resident Physicians’ Understanding of Child Maltreatment: Impacts, Recognition, and Response

**DOI:** 10.3390/ijerph19063319

**Published:** 2022-03-11

**Authors:** Megan Laupacis, Anita Acai, Harriet L. MacMillan, Meredith Vanstone, Donna Stewart, Gina Dimitropoulos, Melissa Kimber

**Affiliations:** 1 Department of Pediatrics, McMaster University, Hamilton, ON L8S 4K1, Canada; megan.laupacis@medportal.ca (M.L.); macmilnh@mcmaster.ca (H.L.M.); 2 McMaster Children’s Hospital, Hamilton, ON L8N 3Z5, Canada; 3 Department of Psychiatry & Behavioural Neurosciences, McMaster University, Hamilton, ON L8S 4K1, Canada; acaia@mcmaster.ca; 4 Offord Centre for Child Studies, McMaster University, Hamilton, ON L8S 4K1, Canada; 5 Department of Family Medicine, McMaster University, Hamilton, ON L8S 4K1, Canada; vanstomg@mcmaster.ca; 6 Centre for Mental Health, University Health Network, University of Toronto, Toronto, ON M5G 2C4, Canada; donna.stewart@uhn.ca; 7 Faculty of Social Work, University of Calgary, Calgary, AB T2N 1N4, Canada; gdimit@ucalgary.ca

**Keywords:** child maltreatment, medical education, education scholarship, Canada, health professions education, mandatory reporting, family violence

## Abstract

Child maltreatment (CM) is a public health problem with devastating effects on individuals, families, and communities. Resident physicians have varied formal education in CM, and report feeling inadequately trained in identifying and responding to CM. The purpose of this study is to explore residents’ understanding of the impacts of CM, and their perceptions of their role in recognizing and responding to CM to better understand their educational needs. This study analyzed qualitative data obtained from a larger project on family violence education. Twenty-nine resident physicians enrolled in pediatric, family medicine, emergency medicine, obstetrics and gynecology, and psychiatry training programs in Alberta, Ontario, and Québec participated in semi-structured interviews to elicit their ideas, experiences, and educational needs relating to CM. Conventional (inductive) content analysis guided the development of codes and categories. Residents had thorough knowledge about the impacts of CM and their duty to recognize CM, but there was less consistency in how residents understood their role in responding to CM. Residents identified the need for more education about recognizing and responding to CM, and the need for educational content to be responsive to training, patient and family factors, and systemic issues. Despite knowledge about the impacts of CM and laws pertaining to mandated reporting, residents reported challenges with responding to concerns of CM. Findings of this study emphasize the need for better training in response to CM. Future educational interventions should consider a multidisciplinary, experiential approach.

## 1. Introduction

Child maltreatment (CM), which includes physical, sexual, and emotional abuse, neglect, and children’s exposure to intimate partner violence (IPV), is an key public health problem that has devastating effects throughout the lifespan [1,2,3,4,5,6]. Research has shown the long-term deleterious effects of CM on relationships, including attachment difficulties, increased risk of IPV victimization, unintended pregnancies, and increased rates of committing violent behaviour, including abuse of one’s own children [1,2,4,5,6,7]. 

CM is a prevalent, global phenomenon. International evidence indicates that over 300 million children experience some form of CM on an annual basis [8]. In Canada, approximately one-third of Canadians will report CM exposure in their lifetime [9,10]; in other countries, the annual and lifetime prevalence of the various forms of CM can exceed 50% [8,11,12]. Globally, research indicates that exposure to CM is associated with cognitive, academic, and employment-related challenges and a range of mental health problems, including suicide attempts [9,13,14,15,16,17]. 

Although common, CM can be challenging to identify by healthcare providers (HCPs), and optimal educational approaches for preparing HCPs to recognize and respond to CM in their clinical encounters are unclear. Importantly, signs and symptoms of CM exposure are not always obvious, and children can be reluctant to reveal information about CM for many reasons, including fear of retribution or shame [18,19,20,21,22]. In addition, HCPs consistently express discomfort identifying CM and a desire for further training [23,24,25,26,27,28,29,30,31,32,33]; HCPs have reported specific challenges with identifying less visible forms of CM, including emotional abuse and emotional neglect, initiating conversations with children and caregivers about potential CM, and ensuring private, safe spaces for children to discuss their concerns and experiences. Similarly, guidance from the clinical literature, as well as from the National Institute of Health and Care Excellence [34] and the World Health Organization [35], indicates that once CM is identified, HCPs must respond safely to the concerns, which includes making a report to child welfare authorities (where legally indicated) and ensuring appropriate follow-up referrals. However, several reports have indicated that HCPs feel unprepared to follow this guidance [30,36,37].

Studies exploring physician-perceived barriers to responding and reporting CM have identified a lack of education, lack of institutional policy, difficulty recognizing CM, fear of legal repercussions, negative experiences with reporting CM, and fear of additional harm to the child as obstructions to HCP responses to CM [23,24,25,26,27,28,29,30,31,32,33]. Studies involving physicians have concluded that despite knowledge of mandated reporting by HCPs, there is confusion about whose responsibility it is to report CM [38,39,40,41], with some physicians believing reporting is the responsibility of a social worker or a physician specializing in CM, which may lead to deferrals or delays in reporting [41]. Identification may be further impeded by personal biases about a family’s education level and race [23,25,42,43]. Outcomes may also be influenced by these factors, with Black and Indigenous children in Canada more likely to be transferred to ongoing services and placed in ‘out of home’ care following a report to child protection services (CPS) [42,43].

It is important to teach resident physicians to recognize and appropriately respond to CM to be able to provide this important form of care as future independent practitioners. This educational imperative has been amplified during the coronavirus disease 2019 (COVID-19) pandemic. Public health measures for reducing the spread and impacts of COVID-19 have exacerbated risk factors for CM; these risk factors include economic uncertainty and instability due to employment layoffs, limited access to health care and social services, as well as reduced availability of support systems, including friends and relatives to support families with childcare [44,45]. Rigorous evidence to corroborate these concerns on a global scale is only just emerging [46]. To this end, there is an urgent need to identify the barriers and facilitators to effectively prepare resident physicians for clinical encounters where CM is an issue. This is especially important as healthcare and social services begin to resume practicing pre-pandemic delivery models and levels of care, and the frequency with which residents encounter children who are at risk for maltreatment or for whom maltreatment is a concern, is likely to increase. In addition, there is evidence that CM training is not consistently and adequately provided to resident physicians. Importantly, physicians and residents who report no specialized training in CM perform worse on tests of knowledge concerning CM [47,48], and may have higher rates of underreporting of CM [49]. Where formal medical education on CM is offered, there is high variability regarding the content, depth, and format of this training across and between residency programs [47,48,50]. The variability in education raises questions about the optimal approach for education on CM for resident physicians and their programs. 

Given the important role that resident physicians have in supporting efforts to prevent CM recurrence and its associated physical and mental health impairment, as well as previous research pointing to the potential influence of attitudes and training experiences on HCP readiness and competence for recognizing and responding to CM, the objectives of this study are twofold. First, this study explores how Canadian residents in specialties where CM is regularly encountered describe their understanding about the impacts of CM, and residents’ perceived role in recognizing and responding to CM. Second, it explores what factors (barriers and facilitators) influence residents’ recognition of and response to CM. Understanding how residents currently perceive their role and what barriers and facilitators they identify in enacting that role is essential for building relevant and responsive medical education opportunities within and across contexts.

## 2. Materials and Methods

### 2.1. Design, Recruitment, and Participants

This study reports an analysis of data obtained from a larger study conducted using qualitative description [51,52]. Qualitative description is a qualitative health research design which is optimal for studies with an emphasis on answering research questions that have applied clinical relevance for healthcare practitioners, educators, and policy makers [53]. It is well suited for the present study given its focus on coalescing a factual summary of phenomena from a purposeful sample of participants; it encourages an analytical approach that stays close to the data, uses low inference, and which dwells on practical relevance of the information for healthcare providers, educators, and policy makers. Further details of the design, recruitment and data collection for the primary study are reported elsewhere [54]. Briefly, data for the larger study were collected from a sample of social work students, social workers, resident physicians, and practicing physicians recruited for Phase 1 of the Researching the Impact of Service provider Education (RISE) Project (riseproject.mcmaster.ca), which aims to determine provider learning needs and preferences related to recognizing and responding to family violence, including child maltreatment and intimate partner violence. The RISE Project was reviewed and approved by the Hamilton Integrated Research Ethics Board (Project #9410), the McGill University Research Ethics Board (Project #20-06-038) and the Conjoint Faculties Research Ethics Board at the University of Calgary (Project #20-0338).

In line with methodological guidelines for qualitative inquiry, participants were sampled and recruited for the RISE Project using non-probabilistic, purposeful sampling procedures [55,56]. Criterion and snowball sampling methods were operationalized via the distribution of recruitment materials through email listservs of (a) six national-level organizations representing trainee and practicing physicians and social workers; (b) residency and social work training programs located in the provinces of Alberta, Ontario, and Québec; (c) professional networks affiliated with RISE Project team members; and (d) participant referrals to the study. Interested individuals were invited to contact the research coordinator to obtain more information, and if applicable, provide consent. A total of 102 individuals participated in Phase 1 of the RISE Project. Eligible participants for the current study (1) were 18 years of age or older; (2) were resident physicians currently enrolled in residency programs in emergency medicine, family medicine, obstetrics and gynecology, pediatrics, or psychiatry located in Alberta, Ontario, or Québec; (3) had provided direct clinical care to patients at least one day per week over the last year; and (4) had the ability to be interviewed in English or French. The present study only includes data provided by residents pertaining to CM. Data sufficiency for the RISE Project and the present study was determined via the construct of information power, which informed our assessment of the sample needed based on the research questions, the homogeneity of the participants on relevant features of interest, the use of theories and frameworks to inform the question and analysis, the quality of dialogue, and the medium focus of the research questions [55]. 

### 2.2. Data Collection

Data were collected via semi-structured one-on-one interviews conducted by trained research assistants via Zoom. Interviews were conducted virtually via Zoom given the ongoing university-based requirements to limit in-person research procedures to reduce the spread and impact of COVID-19 pandemic. The interviews explored how participants think and learn about CM and intimate partner violence, and educational and training needs and preferences related to CM and intimate partner violence. Interviews were completed between 11 July 2020 and 11 December 2020, and were, on average, 46 min in length (range 27 min to 75 min). Data used in the present study comprised approximately one-third of the interview time with participants. Each interview was audio-recorded and transcribed verbatim for data analysis. Members of the interview team met regularly to review consistency in interviewing procedures. These meetings were guided by the research objectives, the expertise of the qualitative leads on the study team (M.K., M.V., and G.D.) and data collection memos generated by the research assistants. Specifically, research assistants were asked to write reflexive memos following each qualitative interview, which were then summarized in batches and shared with the qualitative leads for review and discussion. Table 1 includes the sub-set of semi-structured interview questions relevant to the present study’s research objectives and analyses. For the full interview guide, please contact the corresponding author. 

### 2.3. Data Analysis

Data analysis involved conventional (inductive) content analysis [57,58]. An analytic codebook was developed using an inductive approach via memoing and consensus-based discussion following the independent review of multiple transcripts by members of the research team (M.L., A.A., and M.K.). Iterative readings of the transcripts allowed the identification of key concepts. Concepts were then clustered and reported as discrete categories. Concepts and their overarching categories were verified via triangulated, independent double-coding of six transcripts by the RISE interim research coordinator (A.A.) and reviewed with the RISE Project’s principal investigator (MK), both of whom are PhD-trained researchers with extensive qualitative research experience. The finalized codebook was then applied to all transcripts by the primary author (M.L.; 4th-year medical resident in paediatrics) using line-by-line coding, which helped identify key concepts. A consensus-based discussion within the analytical team ensured consistent application of the codebook. Analysis was managed through the program NVivo [59].

### 2.4. Methodological Rigour

The rigour and trustworthiness of our procedures and findings were promoted using multiple strategies recommended in the methodological literature [60,61]. Specifically, credibility of the research data was enhanced through the generation of multiple data sources (interviews and field memos) and the collection of data from multiple participant types (resident physicians of various specialties). Transferability was promoted via the recruitment of participants located in three Canadian provinces that have varied legal, health, and social responses to CM. Dependability of our analytical procedures was promoted via the use of multiple analysts and dual coding procedures. Other strategies to promote the trustworthiness of our project findings included the engagement of researchers with known expertise and credibility in the field of child maltreatment and qualitative research methods, the maintenance of analytical memos by the primary analysts (M.L. and A.A.), as well as the use of thick, rich description of our research findings that were supplemented via illustrative quotes from an array of research participants.

## 3. Results

### 3.1. Demographics

Table 2 describes the demographics of the 29 resident physicians who participated in this study. Residents included were in emergency medicine (*n* = 6, 20%), family medicine (*n* = 5, 17%), obstetrics and gynecology (*n* = 4, 14%), pediatrics (*n* = 7, 24%), and psychiatry (*n* = 6, 20%) programs. Most residents were women (*n* = 22, 75.9%), and practicing in an urban centre (*n* = 25, 85.2%). The majority of participants (*n* = 16) described having ‘medium familiarity’ with CM, meaning that the participant reported some practical experience related to CM, but did not report it as a focus of their practice. A total of nine participants had a ‘high familiarity’ with CM, indicating they had a lot of practical experience related to child maltreatment and/or described CM as a central focus of their practice. The remaining participants (*n* = 4) had limited or no experience with CM in their clinical practice. 

The data were grouped into three categories: knowledge and ideas about the impact of CM, residents’ perceptions of their roles in recognizing and responding to CM, and barriers and facilitators to recognizing and responding to CM. The table included in the Supplementary File provides more details about concepts and categories and illustrative quotes.

### 3.2. Impact of CM 

Participants’ knowledge and ideas about CM mainly centred on CM impacts for the individual, most commonly the negative psychological impact of CM exposure and its negative effect on the development of future relationships. Many participants considered the psychological and developmental impacts of CM to be longer-lasting than the physical impacts, expressing the view that safety is a prerequisite to growth, development, and social skills. Developmental delay, behaviour problems, and mental health conditions including depression, anxiety, post-traumatic stress disorder, and substance-related disorders were frequently referred to as examples of the long-lasting psychological impacts of CM. Participants described a relationship between CM, IPV, and children’s exposure to IPV, often identifying CM as an adverse childhood experience, and noting that this can increase a person’s likelihood to commit or experience IPV in adulthood. In addition, participants described the impact of CM by a caregiver on children’s’ attachment, confidence, and trust, all of which were described as impacting future relationships, including one’s children, partners, and HCPs:

*It [CM] fundamentally shifts the way that people relate to other people in their sense of security, of safety, ability to trust, what it means about them and their own view or understanding of themselves that they are someone who has been treated that way … I think it often plays out in re-enactments in adulthood and abusive relationships … a belief that they’re someone who deserves to be treated that way or that they don’t know any other way of relating or being in a relationship.*

(Participant 402, Psychiatry)

Participants described the personal emotional toll of learning about CM experienced by their patients. Fear of missing CM, helplessness, not knowing what happens after reporting, and the emotional distress of interacting with a caregiver who was believed to have committed the violence were described by participants as distressing.

*It was hard for me because I’m still trying to treat you like the parent of my patient who is very unwell, but you also might’ve been the one who inflicted this injury. It was a really difficult time for me … feeling this profound feeling of helplessness and not being able to do anything.*

(Participant 301, Paediatrics)

### 3.3. Residents’ Role in Recognizing and Responding to CM

Residents consistently described their primary role as recognizing CM. However, participants had differing perspectives as to what was within residents’ scope of practice in responding to CM. When CM was suspected or disclosed in a clinical encounter, residents indicated that they frequently discussed next steps with their preceptors. Residents articulated that ensuring a child’s physical safety was part of their role. This could include assessing whether there were other children in the home, hospitalizing the child, or making a safety plan with CPS and families. Referring to other individuals (e.g., social workers, specialized CM teams) or outpatient resources was seen as an important aspect of residents’ roles, although this was difficult at times due to lack of access to resources, especially in the outpatient setting. All participants spoke about their knowledge and duty surrounding mandated reporting. Most saw it as their role to make a report to CPS; however, many noted that the responsibility of reporting to CPS was often deferred to preceptors or other HCPs, such as social workers, psychiatrists, or specialized CM teams. Nonetheless, many residents wanted to be more knowledgeable about CM and be able to respond more holistically:

*I don’t have the training or knowledge to intervene farther beyond engaging with social work or talking to psychiatry… I think at least trying to put the wheels in motion or trying to reach out to someone who actually knows what they’re doing or knows a little bit more than us is what we feel obligated to do.*

(Participant 203, Emergency Medicine)

Some participants contrasted CM with IPV, and expressed the view that having a legal obligation to report CM made things more straightforward relative to responding to IPV:

*Knowing about CPS and how easily accessible they are by a phone call, I think that that actually makes it a little easier, perhaps, because I think that there’s that very clear first step of where to go.*

(Participant 509, Family Medicine)

In contrast, participants also described their fear that mandated reporting might cause more harm to the child than not reporting and contribute to the risk of further maltreatment. Examples of possible harms described included terminating or negatively impacting the therapeutic relationship with the family, a potential for increased violence, and possible negative consequences of CPS removing children from their homes. Participants also discussed concerns about a lack of action from CPS and the distress of not knowing what happens after making a report to CPS:

*We end up feeling really handcuffed in some ways … options are that they stay in the home, or we apprehend them … aren’t really actually addressing the issues that are continuing the problem… So, then we start to wonder, did we even do the right thing in the first place, or have we made a bad situation worse?*

(Participant 402, Psychiatry)

Some participants explained that their fear about the possibility of causing harm was particularly salient in reference to equity-deserving populations and given their knowledge of the overrepresentation of Indigenous and racial and ethnic minority children in CM investigations and the disproportionate rates of Indigenous and racial and ethnic minority children being placed in foster care:

*I find that situation [referring to engaging CPS] quite challenging because it completely erodes the trust that the patient may have with the healthcare system. Then I also really struggle with [the] CAS’ [Children’s Aid Society’s] role in marginalized folks’ care and racialized folks’ care knowing that the apprehension rate is significantly higher for Indigenous children and Black children.*

(Participant 603, Obstetrics & Gynecology)

### 3.4. Barriers and Facilitators to Recognizing and Responding to CM

Several barriers and facilitators to residents’ recognition of and response to CM were identified, which can be grouped broadly as clinical encounter factors (including clinical environment and patient/family factors), systemic issues, training, and the roles of other healthcare providers. Participants identified high patient volumes, short visits, and a lack of privacy as environmental factors that create challenges related to safely and efficiently recognizing and responding to CM. Similarly, virtual visits during the COVID-19 pandemic were described as making it harder to recognize CM and to ensure confidentiality.

*Confidentiality is more challenging, and while we do ask that there be a portion of the interview where they can speak confidentially, there’s obviously limits to that if they’re living in the home and someone who might be the perpetrator is actually physically in the same space.*

(Participant 404, Psychiatry)

With respect to patient/family factors, a lack of continuity or prior relationship were described as barriers to CM recognition and response. In contrast, participants indicated that it is beneficial when HCPs have more time with a family, can talk to children or caregivers alone, have pre-existing relationships with families, and can have a continuing relationship after a suspicion of CM is identified. Systemic issues included long wait lists, lack of culturally appropriate care for equity-deserving populations, problems with accessibility (e.g., distance, financial costs, and language), and changes due to the COVID-19 pandemic. 

Participants also described having little formal training on CM as a barrier, and that training was primarily focused on theoretical knowledge, rather than experiential insights. Discomfort with CM, subtlety of presentations, and lack of certainty about a patient’s potential exposure to CM were barriers participants felt may be addressed with more training. These concepts were especially relevant to non-physical forms of violence.

*Yeah, because there wasn’t an overt, ‘The child has been harmed.’ It wasn’t super clear whether the child had witnessed any violence between the parents. But on asking direct question like, ‘Is there any possibility that your child has witnessed these arguments or any violence between the two of you?’ and the patient’s responds, ‘Yes, it’s possible’ and that kind of thing … it didn’t fully sink in that CAS [Children’s Aid Society] involvement was going to be a thing in that until I talked further to my preceptor.*

(Participant 507, Family Medicine)

In contrast, working with preceptors who residents felt were supportive, experienced, and knowledgeable in CM, was identified as helpful to residents in gaining experience and feeling more comfortable in having discussions about CM with families:

*Watching the way that she role modelled an interview with somebody who was vulnerable is something that I’ve carried with me through my practice to this day, five, seven years later.*

(Participant 212, Emergency Medicine)

The most frequently mentioned facilitator to recognizing and responding to CM was working within a team. Participants described gratitude for access to social workers across various settings and teams that specialized in CM, for assistance in navigating the next steps, communicating with families, and collaborating with CPS.

*I’m fortunate that a social worker, she’s integrated in our team … I’m really fortunate that I can text her or call her up whenever I have a case and I’m not really sure how to proceed … I was also able to liaise with other health care professionals involved in the case … so, it all kind of gave me different perspectives, as well as an understanding of each professional’s different roles to play in the care of this patient and their family.*

(Participant 404, Psychiatry)

## 4. Discussion

To better understand the educational needs of residents, this study explored how residents who may encounter CM in their clinical work describe their understanding about the impacts of CM, and their role in recognizing and responding to CM. Resident perceptions of the impact of CM gleaned from this study aligns with the literature [1,2,3,4,5,6,7,9]. Guidance available in Canada and internationally indicates that an important feature of the early and safe identification of CM includes being alert to the signs and symptoms of CM exposure, which includes having an awareness of and being attuned to physical and mental health impairments associated with CM [34,38,62,63,64]. Congruence between what is outlined in those guidance statements and participants’ perceptions about the impacts of CM suggests that, at minimum, residents are receiving some of the foundational information needed to recognize CM in clinical practice. 

Residents in this study reported feeling it was their role to recognize and explore potential signs and symptoms of CM. However, they described that developing this ability requires more training than they had received, especially with recognizing what constitutes CM related to non-physical forms of violence, such as neglect or exposure to IPV. These findings expand what has been reported in the broader literature [30,65,66,67] about resident physicians and CM. They also align with the need to expand the emphasis of training and educational interventions beyond the narrow focus on child physical and sexual abuse [45], as well as the need to consider training and educational approaches that address the possible overlap in the occurrence of IPV and CM, especially in jurisdictions where child exposure to IPV is reportable to child welfare authorities. In doing so, education programs can appropriately and respectfully attend to the safety and wellbeing of children affected by violence, as well as the non-offending caregiver, who is most often the child’s mother. At this point in time, our team is not aware of any educational intervention with empirical evidence demonstrating its ability to improve HCP knowledge, attitudes, skills, and behaviour related to recognizing and responding to all forms of CM in clinical practice; this is a critical gap in health education and clinical scholarship that is both relevant and of interest from a research perspective. Residents also found it more difficult to recognize CM during the pandemic, with increased rates of virtual care precluding physical exams, challenges with confidentiality, and fewer opportunities to observe family dynamics. These challenges are important to address, because clinicians will likely continue to provide some level of virtual care post-pandemic, which may indicate the need for specialized training on virtual interviewing and technology to better ensure privacy.

Residents were more varied in describing their role in responding to CM. Importantly, residents across specialties described struggling with exactly how to report suspected CM, and what happens after the report. Many described that they felt their education stopped at recognition, or at reporting to CPS. In addition, several participants reflected on negative experiences with CPS, with concerns about a lack of action, unequal responses to different families that was felt to potentially be rooted in bias and discrimination, and distress in not knowing the outcome of a report. This is consistent with previous research on experiences with mandated reporting [23,24,25,26,27,28,29,30,31,32,33,39,41], and highlights the need for better understanding and communication between HCPs and CPS about their respective roles. Connecting resident physicians to CPS agencies throughout residency training could foster improved learning and communication in this area. Similarly, including some field experience with local CPS agencies during training may clarify processes of how CPSs follow up with families and may improve understanding, and hence, compliance, with mandated reporting.

Barriers to CM recognition and response were generally consistent with the previous literature [24,25,30,32,33,49]. Knowledge and the availability of local resources may be particularly important to address as a barrier for residents because learners frequently change locations and access to resources may differ depending on location. Residents should be given the opportunity to familiarize themselves with community resources and programs for those experiencing family violence; specific rotations with these programs and services may also offer fruitful learning [34]. At a minimum, program information and lists of resources should be provided in that community. Available guidance notes an ethical and professional imperative to ensuring, as much as possible, the availability of follow-up care that supports the ongoing safety and wellbeing of a child and their family members [34,38,63]. Although this would include any follow-up care that is initiated via mandatory reporting procedures, it also includes ensuring that resident physicians are aware of any local experts who can offer CM assessments, intervention planning, and/or tailored referrals in collaboration with the child and their family. Finally, with residents highlighting systemic issues impacting their patients, it remains vitally important for continued investment in prevention with economic support of families, early childhood education, and parenting skills, all of which can support reductions in CM recurrence and impairment. 

The fear of causing harm was particularly salient in our sample and affirms what has previously been reported by resident and non-resident samples across the globe [30,37,68,69]. Some residents in our sample described this fear in relation to the possibility of contributing to the over-representation of Indigenous and racial and ethnic minority children in foster care, as well as those from families living in communities with high rates of poverty. This has been less frequently reported as a concern of HCPs, despite studies showing disproportionate rates of foster care placements for children from these communities in Canada [42,43], as well as elsewhere around the world [70,71,72,73]. It is encouraging that some residents in this sample were aware of higher rates of CPS involvement and apprehension in these communities, which may reflect greater emphasis recently being placed on the social determinants of health and systemic racism in medical curricula. It may also be reflective of the increased attention on training HCPs in trauma- and violence-informed care (TVIC). TVIC is a model of clinical practice that is resilience-focused and acknowledges that exposure to CM is common, but not a reflection of an individual’s worth, dignity, or potential. A key TVIC principle includes a commitment to safeguard the physical, emotional, and cultural safety of children and their families during clinical encounters; this includes attending to the potential influences of historical and ongoing forms of structural racism and discrimination in healthcare decision making, as well as in CM recognition and response [74]. At minimum, future educational interventions should be careful to address these issues with input and engagement from equity-deserving communities, including determining the relevance of established TVIC models for educational interventions that aim to improve CM recognition and response among resident physicians. Resident education should be sure to involve discussion of CPS contributions toward the disproportionate number of children who are in foster care and from equity-deserving communities. This cannot be achieved without residents being trained on anti-racism, including anti-Indigenous and anti-Black racism, and should be approached in partnership with people from affected communities [75,76]. 

Another barrier that may be especially relevant to learners is a lack of hands-on experience. Residents described discomfort in asking questions about CM and fear of offending or harming patients, with many citing a lack of experience in having discussions about CM. One possible solution is to implement practice sessions, such as a simulation or skills workshops supervised by a preceptor who is experienced in recognizing and responding to CM and related forms of family violence. There is strong evidence that the use of simulation-based methods improves knowledge and skills among undergraduate and post-graduate medical and health professional learners [77,78]. In addition, the education literature offers evidence-based tools that can optimize feedback from preceptor observations and assessments of resident knowledge and skills related to recognizing and responding to CM. Based on our data, we recommend that future research evaluates the integration of this pedagogical evidence with the preferences described by our participants, including the opportunity for residents to: (a) observe preceptor’s role play open communication with families; (b) be involved, when appropriate, in reporting to CPS; as well as (c) coordinate the involvement of follow-up services and support for the family (where there is reason and consent to do so). Relevant outcomes suggested for these evaluations include the assessment of whether these training approaches, compared with those used in usual resident education, improve: (a) resident knowledge and skills for recognizing and responding to CM; and (b) health outcomes for children who have been exposed to CM, which have rarely been considered in the literature. Preceptors should also keep in mind the emotional impact that CM cases can have for learners; this is especially the case when working with learners who may have had personal experience of CM or may be susceptible to vicarious trauma and compassion fatigue, and assist learners in processing these clinical encounters [79,80]. 

Participants consistently expressed the view that children were better served when specialized CM teams were involved. Physicians who have access to expert consultation report feeling more secure in their decisions [30,32], which may be even more important for less experienced learners. A recent and compelling review by Alfandari and Taylor [81] offers important information about the state of the evidence regarding the implementation of multi-professional child protection decision-making teams in hospital-based settings, including their effectiveness, structure, and processes. Across the 26 studies from 10 different countries, the authors reported significant variability in team composition, role structure, and decision-making procedures. In addition, evaluations of team-based decision-making models have centred on CM identification, with no available data on the impacts of team-based decision making on the outcomes for children who have experienced CM and their families; this is an important area for future research [81]. Despite this, the authors offer important recommendations with respect to the need to honour teamwork as a “stand-alone skill that is acquired through education, training, and experience” (p. 15), and which can be fostered using low-stakes pedagogical approaches, such as case-based learning, prior to direct clinical encounters. Collectively, our findings, and those from the review, highlight the merits of fostering interprofessional learning, the need for more evaluation research in this area, and the possibilities of having residents exposed to non-physician preceptors, such as social workers, psychologists, and others as an integral part of residency training in CM recognition and response.

This study was uniquely positioned during the COVID-19 pandemic, and residents reflected that, for many families, risk factors for CM may have been heightened during the pandemic because of increased isolation, unemployment, financial stress, and difficulty accessing mental health and other services. The literature regarding rates of CM during the pandemic is complex, with some preliminary literature showing higher rates of risk factors for CM during the COVID-19 pandemic [82,83,84], and some studies showing reductions in emergency department visits for CM [85,86]. As healthcare services resume typical face-to-face service models and volumes, it would be prudent for researchers to capture and compare CM identification, reporting, and referral practices relative to early and pre-pandemic timepoints, as well as integrate clinical and education findings relevant to CM recognition and response into future clinical, education, and research activities. Pandemic and disaster preparedness plans continue to emphasize strategies for reducing the physiological impacts of viral and extreme events [87]. There is a lack of information about the clinical and education guidance, infrastructure, and processes needed to reduce the broader social and public health impacts of viral outbreaks and disasters, including CM [88,89,90]. However, the need to systematically consider these issues is apparent due to: (a) several epidemics and pandemics occurring over the last two decades; (b) continued public health warnings about the possibility of future catastrophic viral and environmental events; and (c) the strong evidence regarding significant increases in the risk factors for CM in the acute and long-term aftermath of viral outbreaks and disasters. It is possible that evidence-based approaches for safely and effectively recognizing and responding to CM in clinical practice, as well as the substantive education needed for these approaches, do not need to differ prior to, during, or after a pandemic. However, empirical research justifying this statement is needed.

There are important limitations of this study to consider. First, participants’ perceptions of CM were examined, but not IPV, and it is important to note that CM and IPV frequently co-occur. However, child exposure to IPV is considered a form of CM, and participants’ perspectives about this form of CM were included and analyzed in the present study. Second, this study involved participants who volunteered to take part, and therefore may have been more interested in and knowledgeable about family violence. There were considerably more female participants than males, which may reflect women’s greater propensity to participate in interview-based studies [91] and/or the perception that family violence is a “women’s issue.” This study was not intended to be representative of all learners, but intended to provide an in-depth understanding of how this resident sample perceived their roles in relation to CM. It is notable that most residents in the sample (*n* = 16; 55.2%) were characterized by the researchers as having only a medium familiarity with family violence. The sample was relatively junior within residency, with almost half of residents in their first year of training; thus, ideas and concerns shared in this study may be different from those reported by more senior residents in Canada and elsewhere. In addition, the sample was primarily in urban practices; therefore, multidisciplinary teams and experts may have been more readily accessible to them. Data collection occurred within a specific training and geographical context, and the findings may not be transferrable to other health profession education settings or countries. However, several of our findings map onto and expand what has been offered in the existing international literature from healthcare and social service professionals. The purposeful recruitment of medical residents from an array of medial specialties and three Canadian provinces that have varying health, legal, and social responses to CM increases the global transferability of our qualitative research findings. 

## 5. Conclusions

In conclusion, although residents in this study demonstrated good understanding of the impacts of CM and considered recognition of CM as being within their scope of practice, they expressed challenges in knowing how to respond to CM. Future educational interventions should consider hands-on approaches and practice. There is opportunity for education and collaboration with CPS. It would be prudent for residency programs to consider the possibility of rotations with CPS agencies to bolster learning in CM recognition and response, as well as CPS processes for CM investigation and substantiation. Given some of our participants’ negative experiences with CPS, this may foster more understanding and open communication with agencies and their associated providers having expertise in child protection. In addition, resident program development of repositories of local resources that can assist children and families for whom CM is a concern represents an important tool for residents to respond to suspicions and disclosures of CM safely and ethically. Finally, future research should examine the extent to which the availability of multidisciplinary training and child protection decision-making teams supports improvements in resident knowledge, attitudes, skills, and behaviours related to recognizing and responding to CM, as well as patient health outcomes. 

## Figures and Tables

**Table 1:** Semi-structured interview questions.

Interview Section (Pre-Amble)	*Section Questions*
Warm Up	To start, I wonder if you could tell me a little bit about where you are at in your training as a [insert designation]? Please tell me about the most recent practicum/clinical placement you have completed or are currently in.
Main Interview	
Part A: Let’s start by discussing Intimate Partner Violence (or IPV) and Child Maltreatment. By IPV, we mean physical, psychological, sexual or emotional harm by a current or former intimate partner. By child maltreatment we mean physical, sexual, or emotional abuse of a child, as well as neglect. Children’s exposure to IPV between their caregivers has also been increasingly recognized as a form of child maltreatment.	1. How—if at all—have you encountered IPV or Child Maltreatment in your current practice [or practicum] as a [professional designation] in [your current setting]? a. [If participant does not mention CM or IPV] prompt: What about CM/IPV? b. If participant has not encountered either IPV or CM, or says one is not relevant]: Can you tell me why IPV/CM is not seen/not relevant in your current practice? 2. Thinking about what you’ve seen/learned from your current role/practicum training experiences working with [patients/clients], what are the effects of CM as you understand them? a. Clarifying statement if needed: by effects, it’s open to your interpretation, whether it’s on the individual, family, provider; however, you would interpret effects
Part B: Now I want to ask you some questions about how you conceptualize your role when it comes to child maltreatment and intimate partner violence. First, I’m going to ask you some questions about role in recognizing and then I’m going to ask you some questions about your role in responding, if any.	3. As a [professional practitioner/trainee] in your current clinical practice/practicum, do you see it as your role to recognize when one of your clients/patients is experiencing or perpetrating Child Maltreatment? a. If Yes: what specifically is your role? b. If No: Why not? What is your understanding of who this role belongs to? 4. As a [professional practitioner/trainee] in your current clinical placement/practicum, do you see it as your role when it comes to responding to someone experiencing or perpetrating CM? a. If Yes: what specifically is your role? b. If No: Why not? What is your understanding of who this role belongs to? 5. What, from your perspective is the greatest facilitator/barrier for you working in [your professional role/at your practicum setting] to recognize and respond to CM in practice?

**Table 2:** Participants’ demographic characteristics.

Sample Characteristic	*n* (%)
Practice Community	29 (100)
Urban	25 (85.2)
Rural	3 (10.3)
Combined	1 (3.4)
Gender	29 (100)
Woman	22 (75.9)
Man	6 (20.7)
Prefer to self-identify	0 (0)
Chose not to report	1 (3.4)
Residency Year	29 (100)
PGY1 *	14 (48.3)
PGY2	4 (13.8)
PGY3	4 (13.8)
PGY4	4 (13.8)
PGY5	1 (3.4)
Not Reported	2 (6.9)
Province	29 (100)
Alberta	12 (41.4)
Ontario	10 (34.5)
Québec	7 (24.1)

* PGY, postgraduate year (i.e., year of training).

## Data Availability

This dataset is not available for sharing outside of the research team. When participants consented to participating in the study, they did not consent to access to transcripts or data beyond the research team. Some complete qualitative transcripts may be identifiable. Please contact the corresponding author for further clarification.

## Supplementary Materials

The following supporting information can be downloaded at: https://www.mdpi.com/article/10.3390/ijerph19063319/s1, Table S1: Qualitative categories, concepts, codes, subcodes, and exemplar quotes.
