# Supplementary material for: A Qualitative Description of Resident Physicians’ Understanding of Child Maltreatment: Impacts, Recognition, and Response
Source: Int J Environ Res Public Health. 2022 Mar 11;19(6):3319. doi: 10.3390/ijerph19063319 (PMC8949331; doi:10.3390/ijerph19063319)
Supplement: Supplementary file 1 [file ijerph-19-03319-s001.zip › ijerph-1591826-supplementary.pdf]

A Qualitative Description of Resident Physicians' Understanding of Child Maltreatment: Impacts, Recognition, and Response

Supplementary File

Table S1. Qualitative categories, concepts, codes, subcodes, and exemplar quotes

Category	Concepts	Codes & subcodes	Exemplar quote
Knowledge & ideas about impact of CM	Family violence	Relationship between CM & IPV	"It [CM] fundamentally shifts the way that people relate to other people, their sense of security, of safety, ability to trust, what it means about them and their own view or understanding of themselves … and I think it often plays out in re-enactments in adulthood and abusive relationships." Participant 402, Psychiatry
	Impact	Individual<br>Psychological<br>Relationships<br>Physical<br>Developmental	"We see that in the adult population … many patients have PTSD, end up homeless, often involved in like alcohol use disorder or opioid use disorder as a way of coping. And when you speak to them, many times there has been a lot of trauma in their childhood." Participant 204, Emergency Medicine
		Provider	"Of course, it's difficult for us to see this, and to realise that somebody is experiencing this, and to know that somebody is doing this to somebody else, sometimes is unimaginable… it also changes, based on the evolution, and one's experience and our experience if we have children, children of the same age group, for families who experience the same thing. I think it can also affect us." Participant 201, Emergency Medicine
		Community	"I think there are broader societal effects, of course, in terms of how we care for those and provide support for those who are experiencing either CM or IPV… not just from a healthcare provider setting but obviously from community supports and social supports and politics and legal and law enforcement." Participant 302, Pediatrics
Role	Recognition	Recognition	"To me, all the skills I need to acutely recognize and manage it are kind of what matter more so than the kind of long-term effects of it [CM]." Participant 210, Emergency Medicine
	Response	Scope of practice	"I don't think all physicians need to be experts in management long-term of PTSD [post-traumatic stress disorder] or psychosocial issues relating to IPV… or completely navigating the Children and Family Services system … But I think, should everyone in City X be able to name a women's shelter … Should everyone be able to know what needs to be reported to Children's Services? Yes. So, I think it's hard to define what that scope is, but I think there's certain things that everyone needs to know, and then there are certain things that are kind of consultant or expert-level knowledge." Participant 210, Emergency Medicine
		Support	"They were really worried that … they were going to be accused and the baby was going to be taken away. So, we sat down together, and we tried to address all of that. I tried to make them

		Advocacy<br>Safety<br>Resources	understand that [the] CAS [Children's Aid Society] is ultimately a service that is there for support and is there to help for people that might need assistance in terms of concern about their child's safety… And I felt that it was a really impactful encounter because it was the first time that I had had the chance to really bring to their attention what CAS was and try to voice what child safety could be to a parent." Participant 506, Family Medicine
		Legal obligations / Mandated reporting	"It's one of those things that you learn early in Medical School … Once we've recognized CM, we don't really have an option. I always tell the patient I have to call [the] CAS [Children's Aid Society] because you told me that and then you have to do it." Participant 603, Obstetrics & Gynecology
		Child Protection Services (CPS) attitudes & experiences	"With [the] CAS [Children's Aid Society] up until recently we had a binder where alerts would exist on the [labour and delivery] ward from CAS about certain parents. When the child was born, we would know if there had already been an alert out from CAS and potentially what the plan may be so whether that is apprehension or support … I find it really challenging for me, personally, when there's a bit of what I always feel is deceit in a way where even if we know the plan is for apprehension, you're not allowed to tell the parents that that's the plan because that may lead to the parent fleeing prior to the delivery of the child and could put the apprehension in jeopardy." Participant 603, Obstetrics & Gynecology
		Others' roles	"If I am concerned that CM is possibly at play or even that there's IPV within the home, my first step is always to consult with my social work colleague, and from then on, to kind of devise a plan of how we move forward. Oftentimes, in order to preserve the therapeutic alliance with the family and the patient, we'll typically have the social work colleague be more explicit in addressing issues around CM or IPV. Of course, depending on the consultation of my social work colleague, that's when we might signal to Youth Protection Services, sometimes consult with them on anonymous grounds, and get greater guidance or clarity. But I think there's a role in terms of collaboration." Participant 404, Psychiatry
Barriers	Clinical encounter	Environment<br>COVID-19	"You have 15 minutes. You're sort of in and out. 'Give me your one thing we need to talk about, and then we'll manage it,' and trying to get people out the door because the next person is coming in, and we're trying not to have everyone piling up in the waiting room at the same time. I think that really sort of challenges those conversations." Participant 509, Family Medicine
		Patient / family factors<br>Dependence on	"The pediatric population is a lot more vulnerable in that they can't always speak up for themselves. They don't have the resources or know that something might be right or wrong." Participant 308, Paediatrics

		others<br>Relationship with Provider	
	Systems issues	Fear of causing harm<br>Bias	"I also really struggle with [the] CAS' [Children's Aid Society's] role in marginalized folks' care and racialized folks' care knowing that the apprehension rate is significantly higher for Indigenous children and Black children. What am I setting up these families for when CAS gets involved and I think that's something that I definitely struggle with." Participant 603, Obstetrics & Gynecology
		Subtlety / unsureness	"Nobody comes into a clinic where their reason for consultation is like, 'Rule out CM.'" Participant 309, Paediatrics
		Resources	"The biggest roadblock that I find as a resident is trying to understand community services and outreach programs that can truly assist individuals that face IPV or children that are in such situations." Participant 403, Psychiatry
	Training	Hierarchy	"As a resident trainee, I think that's something that always comes with a bit of caution in the sense that I don't have the full authority of the staff for a patient that I'm treating to make strong judgment calls about maltreatment. I can suggest it, I can bring it up to the staff, but I can't necessarily… I don't think I can approach it with as much assertiveness as a staff would simply by the status of my ranking." Participant 506, Family Medicine
		Scope of practice	"I don't have the training or the knowledge to intervene farther beyond engaging with social work or talking to psychiatry." Participant 203, Emergency Medicine
		Lack of education / experience	"We really don't have much training and education on this. They are rather theoretical concepts. With such-and-such of a fracture, you must consider this … it's very theoretical and very little related to our practice." Participant 207, Emergency Medicine

Facilitators	Clinical encounter	Environment	"Like I'm lucky to be at Medical Practice X where we have… like there's video cameras in every room, where if I'm seeing somebody as a resident, usually my preceptor will try to watch me while I'm doing that so that they can give me feedback." Participant 507, Family Medicine
		Protocols	"Having something that you can reference on shift where, for example, we have certain clinical pathways. Someone comes in with this presentation and it's in the chart or you go down this pathway of yes, no, X is the treatment, Y is the treatment." Participant 203, Emergency Medicine
		Relationship with provider / continuity	"Because I was working with a patient and family across multiple clinical encounters, I was able to develop a certain alliance." Participant 404, Psychiatry
	Other's roles	Teams	"There is a whole list of individuals who can interact with a patient regarding their health—nurse practitioners, counsellors … there's probably a different degree of understanding of the patient's social situation in each of these roles … I think that sort of an interprofessional team approach where everybody sort of feels a sense of responsibility is really important." Participant 509, Family Medicine
		Legal / CPS involvement	"I don't really have a choice. I know at least an agency that I have to engage. It feels more clear cut what I have to do." Participant 603, Obstetrics & Gynecology
	Training	Experience	"I think the trial and error that I went through with that case was a meaningful experience in the sense that I learned how to navigate the system. I also learned that my suspicions were valid and that I did the right thing." Participant 212, Emergency Medicing
		Mentors	"I think that most of my learning on this that's been very impactful has been when I come across cases and talking to my staff and my preceptors and getting some of their experience passed on to me." Participant 608, Obstetrics & Gynecology
